# Krüppel-like Factor 4 modulates interleukin-6 release in human dendritic cells after *in vitro* stimulation with *Aspergillus fumigatus* and *Candida albicans*

**DOI:** 10.1038/srep27990

**Published:** 2016-06-27

**Authors:** Kristin Czakai, Ines Leonhardt, Andreas Dix, Michael Bonin, Joerg Linde, Hermann Einsele, Oliver Kurzai, Jürgen Loeffler

**Affiliations:** 1 University Hospital of Würzburg, Department of Internal Medicine II, WÜ4i, Würzburg, Germany; 2 Septomics Research Centre, Friedrich Schiller University and Leibniz Institute for Natural Product Research and Infection Biology-Hans-Knöll-Institute (HKI), Jena, Germany; 3 Systems Biology/Bioinformatics, Leibniz Institute for Natural Product Research and Infection Biology – Hans-Knöll-Institute, Jena, Germany; 4 Institute of Medical Genetics and Applied Genomics, University of Tübingen, Tübingen, Germany

## Abstract

Invasive fungal infections are associated with high mortality rates and are mostly caused by the opportunistic fungi *Aspergillus fumigatus* and *Candida albicans*. Immune responses against these fungi are still not fully understood. Dendritic cells (DCs) are crucial players in initiating innate and adaptive immune responses against fungal infections. The immunomodulatory effects of fungi were compared to the bacterial stimulus LPS to determine key players in the immune response to fungal infections. A genome wide study of the gene regulation of human monocyte-derived dendritic cells (DCs) confronted with *A. fumigatus, C. albicans* or LPS was performed and *Krüppel-like factor 4 (KLF4)* was identified as the only transcription factor that was down-regulated in DCs by both fungi but induced by stimulation with LPS. Downstream analysis demonstrated the influence of KLF4 on the interleukine-6 expression in human DCs. Furthermore, KLF4 regulation was shown to be dependent on pattern recognition receptor ligation. Therefore KLF4 was identified as a controlling element in the IL-6 immune response with a unique expression pattern comparing fungal and LPS stimulation.

Dendritic cells (DCs) play an important role in pathogen recognition. They recognize pathogens via pattern recognition receptors (PRRs) that are intracellularly localized or expressed on the cell surface^1^. Due to biochemically diverse compositions of the pathogen associated molecular patterns (PAMPs) such as LPS, β-glucan, or peptidoglycans, antigen presenting cells (APC) are able to discriminate between a large variety of pathogens^2^,^3^. The Toll-like receptors TLR2, TLR4 and the C-type lectin Dectin-1 are the main receptors involved in the recognition of fungal pathogens^4^. Upon pathogen recognition by these receptors, a number of intracellular signalling pathways including NF-kB, PI3K and MAPKs are activated in DCs and characteristic phenotypic and functional changes involved in the process of DC maturation are induced^5^. Furthermore, depending on the recognized pathogen, the regulation of transcription factors also influences the expression of specific pro- and anti-inflammatory cytokines. Transcriptional analysis was used to identify key fungus specific transcription factors in the immune response of DCs stimulated with *A. fumigatus* and *C. albicans* compared to the bacterial control antigen LPS. Those fungi were selected as invasive aspergillosis and invasive candidiasis are the most common invasive fungal infections in patients with haematological malignancies^6^.

The transcription factor Krüppel-like factor 4 (KLF4) was identified as important player in the immune response to fungal infections. KLF4 has a variety of biological functions and previous work on this transcription factor has demonstrated its roles in both, the development of inflammatory monocytes and in the production of inflammatory cytokines^7^,^8^, thereby KLF4 displayed a pleiotropic nature, as demonstrated by its ability to either activate or repress transcription, depending on the context^9^. In a murine system an influence of KLF4 on the Th2 response and on IL-6, a cytokine with pleiotropic functions and plays an important role in the transition from innate to acquired immunity and in promoting Th2 response was demonstrated^10^,^11^,^12^. In consequence, we sought to analyse the possible regulatory role of KLF4 in the transition from innate to acquired immunity during fungal infections.

## Results

### Transcriptome analysis of human DCs identifies fungal specific regulation of KLF4

**Figure 1:**
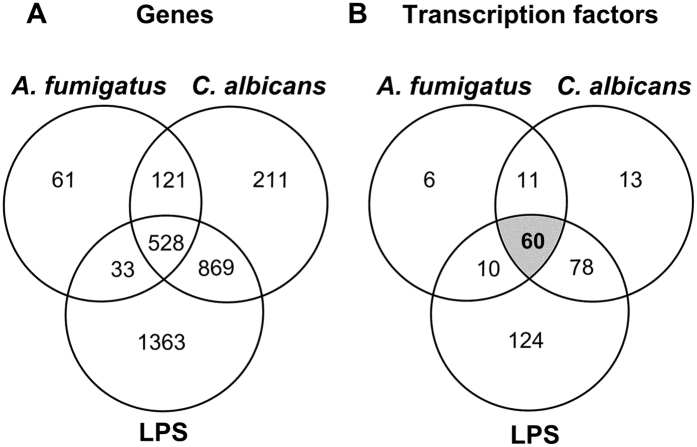
Venn diagram of differentially regulated Genes and transcriptions factors. DCs were either stimulated with *A. fumigatus* (Af), *C. albicans* (Ca) (both MOI 1) or LPS (1 μg/ml) in four independent experiments for 6 h. Affymetrix whole genome expression arrays (HGU-219) were performed and mRNAs with log fold changes >1 and a p-value < 0.01 were considered differentially regulated. (**A**) Regulated mRNAs and (**B**) transcriptions factors were visualized by Venn diagram analysis to identify overlapping expression patterns.

Human DCs were confronted with *A. fumigatus*, *C. albicans* and LPS, which was used as a standard control stimulus. Whole transcriptome analysis was performed at 6 h post infection of four independent biological replicates. Hierarchical clustering analysis was used to examine differences in the response of DCs to LPS and the fungi and revealed a clustering of the *A. fumigatus* and *C. albicans* stimulated DCs (Supplementary Figure S1). Stimulation with LPS induced a differential regulation of 2793 genes after 6 h, while confrontation with *A. fumigatus* and *C. albicans* resulted in 743 and 1729 differentially regulated genes, respectively. 528 genes were regulated in all tested conditions after 6 h of co-cultivation (Fig. 1A).

**Figure 2:**
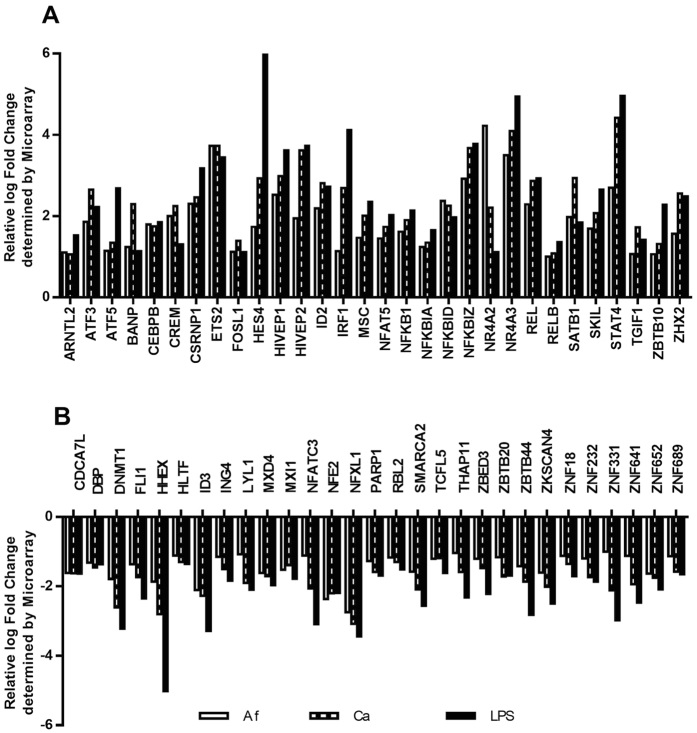
Regulation of transcription factors. DCs were either stimulated with *A. fumigatus* (Af), *C. albicans* (Ca) (both MOI 1) or LPS (1 μg/ml) in four independent experiments for 6 h. Affymetrix whole genome expression arrays (HGU-219) were performed. Displayed are the fold changes of the 59 of 60 differentially regulated transcription factors identified in Fig. 1B that were commonly regulated by *A. fumigatus*, *C. albicans* and LPS. (**A**) displays up- and (**B**) down-regulated transcription factors.

**Figure 3:**
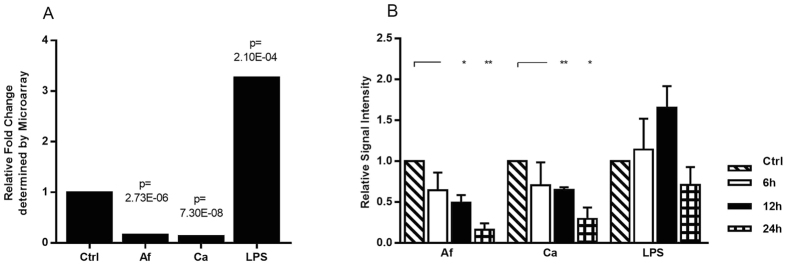
KLF4 was the only transcription factor down-regulated by *A. fumigatus* and *C. albicans* but induced by LPS. DCs were either stimulated with *A. fumigatus* (Af, light shaded), *C. albicans* (Ca, dark shaded) (MOI 1) or LPS (1 μg/ml, black). (**A**) KLF4 mRNA regulation after 6 h was determined by Affymetrix whole genome expression arrays (HGU-219) of four independent experiments. Displayed are the logfold changes with added FDR adjusted p-Values of the differentially regulated gene *KLF4*. (**B**) DC total cellular extracts of three independent experiments, co-cultivated for 6 h (white bars), 12 h (black bars) and 24 h (squared bars) were analyzed by western blotting. The time dependent KLF4 protein regulation was analyzed by ImageJ, relative to untreated control (striped bars) and loading control β-Actin. Significant changes in protein expression are labeled with asterisks (*p < 0.05, **p < 0.01 Student’s paired t-test). Data is illustrated as mean plus standard error of the mean (SEM).

These 528 genes were used for an analysis of regulated transcription factors to get a better insight in cellular responses of DCs facing either fungi or LPS and to determine differences in the regulation pattern between fungi and LPS. A total of 1896 genes are listed as transcription factors in the TRANSFAC database^13^, which is a knowledge-based database of published eukaryotic transcription factors (www.biobase-international.com) and 60 transcription factors were differentially regulated among the 528 genes (Fig. 1B). 59 Transcription factors (Fig. 2) were either induced or reduced by all three stimuli. *KLF4* was identified as the only transcription factor that showed a different regulation pattern comparing bacterial and fungal stimuli (Fig. 3A). Expression of *KLF4* in DCs was strongly reduced during contact with *A. fumigatus* (log fold change: −2.6, *p*-Value = 2.73E-08) and *C. albicans* (log fold change: −2.9, *p*-Value = 1.48E-09) whereas LPS stimulation induced a 1.7 log fold up-regulation (*p*-Value = 4.38E-05). This differential regulation was validated by RT-qPCR on RNA isolated from DCs stimulated with *A. fumigatus, C. albicans* and LPS for 6 and 12 h which showed a strong down-regulation of *KLF4* in fungal treated DCs (not shown). In contrast, LPS stimulation induced *KLF4* gene expression after 6 h; this induction was time-dependent and not stable over 12 h. Furthermore we analyzed the regulation of KLF4 by fungi and the bacterial control stimulation at the protein level after stimulation for 6, 12 and 24 h (Fig. 3B). A time-dependent reduction of KLF4 protein in DCs stimulated with either *A. fumigatus* or *C. albicans* was observed and significant after 12 and 24 h co-cultivation. The control stimulus LPS induced up-regulation of KLF4, which was confirmed for the time points 6 and 12 h but was not stable over 24 h.

### KLF4 is involved in the IL-6 response

One of the main functions of DCs is to act as a bridge between the innate and adaptive immune system. KLF4 was identified by GOrilla analysis to be a member of Gene Ontology categories such as response to stress (GO: 0006950), regulation of programmed cell death GO: 0043067), regulation of signal transduction (GO: 0009966) and regulation of cytokine production (GO: 0001817). To determine the biological consequence of KLF4 reduction by fungi, the culture condition with induced KLF4 expression level was taken for further downstream analysis using RNA interference gene knock down. For these experiments DCs were electroporated with KLF4 siRNA and random, non-silencing (ns) siRNA treated cells served as controls. siRNA transfection resulted in a >75% KLF4 transcript reduction compared to the non-silencing control (supplementary Figure S2A). In addition, knock-down of KLF4 by siRNA was also confirmed at protein level (supplementary Figure S2B).

**Figure 4:**
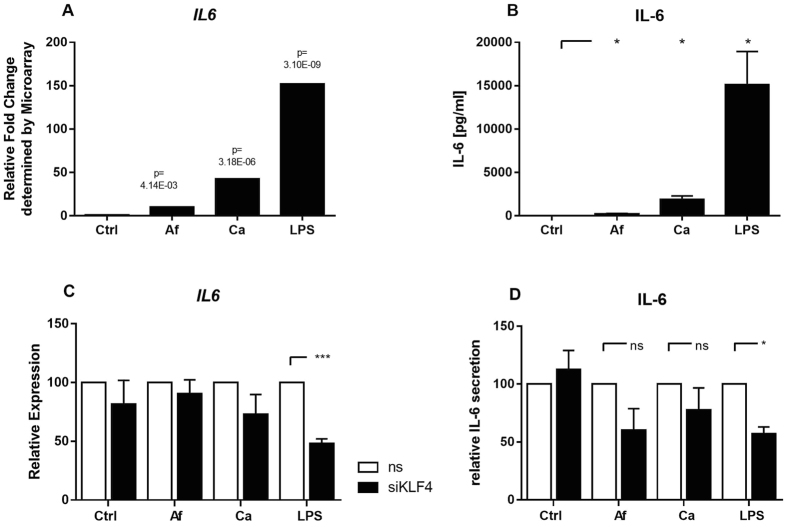
KLF4 dependent IL-6 regulation. DCs were stimulated with LPS (1 μg/ml) *A. fumigatus* (Af) *C. albicans* (Ca) (both MOI 1) or left untreated for 6 h. (**A**) IL6 mRNA regulation was determined by microarray analysis. (**B**) IL-6 secretion was quantified by enzyme linked immunosorbent assay. (**C,D**) DCs were transfected by electroporation with either non-silencing siRNA (white bars) or with siRNA targeting KLF4 (black bars). 24 h after electroporation, DCs were stimulated with *A. fumigatus*, *C. albicans* (MOI1) and LPS (1 μg/ml) for 6 h. (**C**) IL6 mRNA level were quantified by real-time PCR relative to non-silencing control. ALAS1 served as reference gene. (**D**) Release of IL-6 of LPS stimulated DCs was quantified by enzyme linked immunosorbent assay. Data is illustrated as FC or mean plus SEM of 3 to 4 independent experiments (*p < 0.05, **p < 0.01, ***p < 0.001; Student’s paired t-test).

To identify down-stream molecules of the transcription factor KLF4, we conducted a literature research and identified CCL2, RANTES, CXCL10, TNF, IL-10, IL-1 and IL-6 as potential immune relevant targets of KLF4^14^,^15^,^16^,^17^,^18^,^19^,^20^. In our experiments, we did not detect any KLF4-dependent regulation of IL10, RANTES, CXCL10, IL1B, and CCL2 but a significant influence on TNF (supplementary Figure S3). However, IL6 regulation was highly affected by the KLF4 knock-down. While KLF4 silencing in fungal-stimulated DCs weakly reduced IL6 expression, IL6 levels were significantly reduced in LPS treated control DCs, compared to the non-silencing control (51,8%, p-Value = 0.009) (Fig. 4C). Furthermore, the LPS-stimulated and KLF4-siRNA transfected DCs secreted significantly reduced amounts of IL-6 compared to control (42,8%, p-Value 0.0175) (Fig. 4D). This confirmed the gene expression data and showed a KLF4 dependent regulation of IL-6 in DCs at the level of both RNA and protein. Furthermore, no siKLF4 dependent change of the cytokine levels of TNF-α, IL-1α, IL-1β, IL-10, IP-10 (CXCL10), MIP-3α (CCL20), MCP-1 (CCL2), MIP-1α (CCL3) and RANTES (CCL5) was detected (supplementary Figure S4). No secretion of IFN-gamma, IL-12p70, IL-15, IL-18 and IL-2 was detectable (not shown). This confirms the specificity of KLF4 on the IL-6 regulation. After incubation of non-electroporated DCs with *A. fumigatus*, *C. albicans* or LPS *IL6* regulation and IL-6 release was significantly increased by stimulation with fungi and LPS (Fig. 4A+B). However, the LPS induced IL-6 release was considerably more pronounced compared to fungi.

### KLF4 regulation is dependent on PRR activation

**Figure 5:**
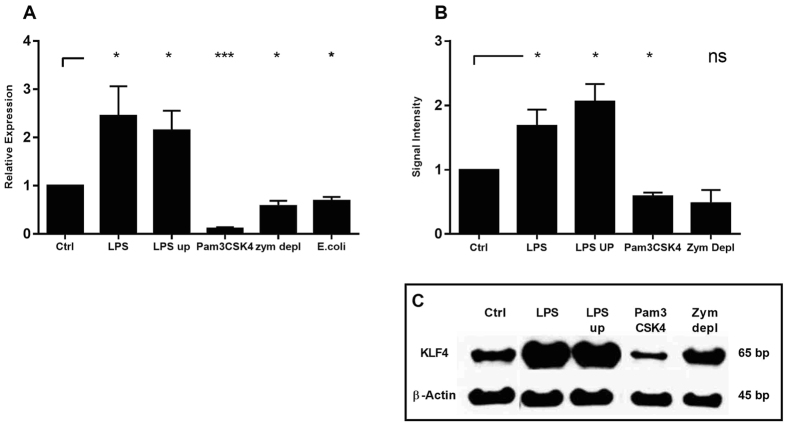
KLF4 regulation is dependent on PRR activation. DCs had been co-cultivated with LPS, LPS ultra-pure (LPS up) (1 μg/ml), depleted zymosan (100 μg/ml), Pam3CSK4 (100 ng/ml) or *E.coli* (MOI1). (**A**) After 6 h of co-cultivation mRNA was isolated and *KLF4* mRNA was quantified by quantitative real-time PCR relative to untreated control (Ctrl). ALAS1 served as reference gene. (**B**) Western blot of KLF4 was performed of DCs cell lysates co-stimulated for 12 h. Data was analyzed relative to untreated Control (Ctrl). β-Actin served as loading control (*p < 0.05, ***p < 0.001 Student’s paired t-test). Data of five independent experiments is illustrated as mean plus SEM. C) Exemplary Western Blot.

Activation of human DC is mediated via PRRs. To further investigate which PRRs are involved in differential KLF4 regulation, we stimulated DCs with defined ligands of several PRRs. We used LPS, a known TLR4 and TLR2 ligand and ultra-pure LPS, which exclusively interacts with TLR4. The synthetic triacylated lipoprotein Pam3CSK4 was used for the activation of TLR2/TLR1 signalling and depleted zymosan, a β-glucan, for Dectin-1 activation. All of these PRRs have been shown to be involved in fungal – DC recognition and activation. Additionally, DCs were co-cultivated with E.coli and A. fumigatus conidia to analyse if phagocytic mechanisms might affect regulation of KLF4. Stimulation of DCs with ultrapure LPS and LPS led to increased expression of KLF4. However, stimulation with the TLR2/TLR1 and Dectin-1 ligands Pam3CSK4, depleted zymosan significantly decreased KLF4-expression in DCs (Fig. 5A). These transcription data were further confirmed by western blot analysis (Fig. 5B). At the protein level only depleted zymosan and Pam3CSK4 reduced KLF4 protein. This indicates a PRR-dependent distinction in KLF4 signalling indicating TLR4 activation to be responsible for KLF4 accumulation and TLR2 and Dectin-1 for reduction of KLF4. Stimulation with E coli significantly reduced KLF4 expression in DCs. However, no KLF4 regulation was observed in DCs stimulated with A. fumigatus conidia that are also internalized by DCs (data not shown).

**Figure 6:**
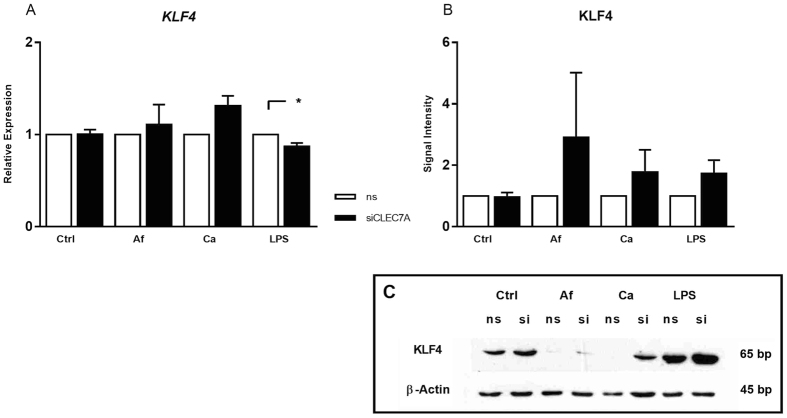
Influence of CLEC7A knock down on KLF4. DCs were transfected by electroporation with either non-silencing siRNA (white bars) or with siRNA targeting CLEC7A (black bars). 24 h after electroporation, DCs were stimulated with *A. fumigatus*, *C. albicans* (MOI1) and LPS (1 μg/ml). (**A**) KLF4 mRNA level were quantified after 6 h by real-time PCR relative to non-silencing control. ALAS1 served as reference gene. (**B**) Western blot of KLF4 was performed of DCs cell lysates co-stimulated for 12 h. Data was analyzed relative to non-silenced control relative to β-Actin as loading control (*p < 0.05 Student’s paired t-test). Data of four independent experiments is illustrated as mean plus SEM. ( ) Exemplary Western Blot.

To further assess the role of the PRRs in KLF4 regulation, RNA interference using siRNA targeting CLEC7A (Dectin-1) mRNA was performed. The siCLEC7A treatment achieved a reduction of CLEC7A mRNA over 90% and on protein level over 80% (not shown). An induction of *KLF4* of DCs stimulated with fungi was observed, while *KLF4* was reduced in siCLEC7A transfected DCs stimulated with LPS (Fig. 6A). On protein level an induction of KLF4 in siCLEC7A treated DCs stimulated with either *A. fumigatus, C. albicans* or LPS was determined (Fig. 6B).

## Discussion

*C. albicans* and *A. fumigatus* are opportunistic fungal pathogens that cause potentially life-threatening diseases in individuals with an immunodeficiency or immunosuppression^21^. The incidence of fungal diseases has increased over the past decades; in hematopoietic stem cell transplant recipients invasive aspergillosis and invasive candidiasis are the most common invasive fungal infections^2^,^6^.

To further characterize the immune response and immunopathology and to identify distinct marker molecules regulated by fungi a genome wide transcriptome analysis was performed. In this study KLF4 was identified as the only transcription factor that showed inverse expression in DCs if co-cultivated with either *A. fumigatus*, *C. albicans, E. coli* or the control stimulus LPS.

Our results confirm data from murine macrophages in demonstrating KLF4 induction in human DCs after LPS stimulation^22^. Interestingly, co-cultivation of DCs with *A. fumigatus* or *C. albicans* led to specific *KLF4* down-regulation. This specific expression pattern was time-dependent as shown at RNA and protein levels. In human macrophages, *KLF4* was induced after six hours of co-cultivation with LPS, while a prolonged stimulation of 16 h lead to down-regulation in murine macrophages^7^,^16^. This implies a role of KLF4 as a mediator of early cellular responses. In a murine model LPS is able to induce KLF4 expression, which in consequence leads to activation of inducible nitric-oxide synthase (iNOS) and nitric oxide, a key mediator in the innate immune defence^7^. This iNOS activation is cooperatively induced with NFκB p65 (RelA) activation. Thus, besides iNOS activation, KLF4 regulates inflammatory signals via TGF-ß1/Smad3 expression, demonstrating its prominent role as a regulator of key signalling pathways^7^. In murine DCs, KLF4 was found to bind to and activate the IL-6 promotor^20^. Moreover, in mouse microglial cells, KLF4 knock-down decreased levels of the pro-inflammatory cytokine IL-6^18^. Consequently, we analysed KLF4-dependent regulation of IL-6 in human DCs by RNAi knock-down of *KLF4*. This knock-down resulted in reduced *IL6* expression levels and reduced IL-6 secretion by DCs. Interestingly, if human DCs were stimulated with fungus, low-level induction of IL-6 was observed, which was influenced by *KLF4* knock-down. Expression of other inflammatory molecules such as *RANTES, IL10* and *CXCL10* were not targeted by *KLF4* silencing, indicating specificity for *IL6* regulation. Also no influence of KLF4 knockdown on cytokine levels of IL-1α/β, IL-10, IP-10 (CXCL10) and MIP-3α, MCP-1 (CCL2), MIP-1α and RANTES was detected. Relevance of silenced KLF4 on TNF alpha levels was only detectable on mRNA but not on protein level.

**Figure 7:**
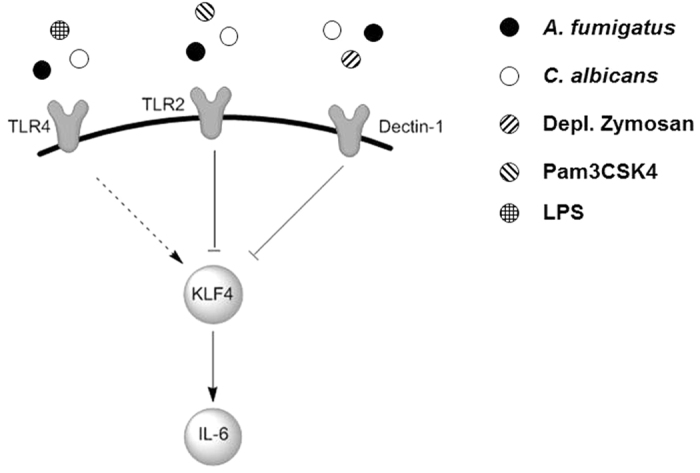
Schematic representation of the PRR specific IL-6 regulation via KLF4.

DCs and other mononuclear phagocytes express PRRs on their surface to enable the recognition of pathogens. TLR2, TLR4 and Dectin-1 are the major receptors on the surface of DCs involved in fungal recognition^23^. TLR2 is mainly known for the recognition of peptidoglycan, the major components of gram-positive bacterial cell wall; whereas TLR4 is activated by LPS, derived from gram-negative bacteria^24^. TLR deficient mice provided evidence for the involvement of both, TLR2 and TLR4 receptors in the recognition of *Aspergillus spp.* and *Candida spp.*^24^, depending on the fungal morphology and cell wall composition, though the main receptor for *A. fumigatus* and *C. albicans* is Dectin-1^25^,^26^. This type II transmembrane protein binds to β-glucan, a major structural fungal cell wall component^26^. We dissected which of these receptors is involved in KLF4 regulation. LPS and LPS ultra-pure are ligands for TLR4/TLR2 and TLR4, respectively. Both ligands induced KLF4 expression. On the other hand, activation of TLR2/TLR1 by Pam3CSK4 or Dectin-1 activation by depleted zymosan reduced KLF4 expression (Fig. 7).

Interestingly, stimulation with *E.coli* also reduced KLF4 gene expression, whereas in contrast, no regulation of KLF4 by stimulation with inactivated *A. fumigatus* conidia was observed. Conidia are covered with a hydrophobic rodlet layer that prevents recognition by PRRs^27^. This gives hint on the involvement of the phagocytic machinery on KLF4 regulation. However, phagocytic events alone are obviously not sufficient to impact KLF4 regulation. In consequence, recognition via PRRs seems to be essential for KLF4 regulation.

To determine downstream effects of the transcription factor KLF4, a large variety of cytokine levels was quantified. Cytokine secretion is a main DC function in the transition from innate to acquired immunity. IL-6 was significantly reduced after *KLF4* knockdown and subsequent LPS stimulation; these effects were less prominent after fungal stimulation. Thus, specific binding of ß-glucan to Dectin-1 activated at least partly alternative pathways, such as CD37 co-localization. The tetraspanin CD37 is important for dectin-1 stabilization in membranes of DCs and controls dectin-1-mediated IL-6 production. Furthermore, both, TLR2 and Dectin-1 collaborate in inflammatory responses and Dectin-1 was shown to amplify TLR2 dependent cytokine induction in mice^4^,^26^. Thus, IL-6 production and secretion might depend on the individual stimulus and thereby KLF4 is a regulator of IL-6 levels. A TLR4 dependent attenuation of IL-6 release by α- and β-glucan and galactomannan and an α-glucan dependent IL-6 response via TLR2 signalling was shown^21^. The TLR signalling effects are dependent on the morphological state as hyphae but not conidia selectively down-regulated the TLR4 response^25^. This may indicate an immune evasion strategy of the fungus by reducing the immune system activation.

In summary, we describe for the first time KLF4 as an important regulatory factor in the host response to fungi and its PRR-dependent regulation. Furthermore, an influence of KLF4 on IL-6 secretion was shown in human DCs. IL-6 is a pleiotropic cytokine that has been implicated in a number of diseases and inflammatory settings and plays an important role in the transition from innate to acquired immunity^10^. At the transcriptional level, it is similarly regulated in response to fungal and bacterial infection, both in DCs and human whole blood^28^. A major function of IL-6 is T-cell recruitment, but IL-6 also takes part in naïve CD4^+^ T-cell differentiation, thereby promoting Th2 and Th17 responses^10^. The Th1/Th2 balance is known to be a critical factor which determines the outcome of invasive fungal infections^2^. KLF4 may influence the Th1/Th2 balancing via IL6 and thereby be a key regulator in fungal infections.

## Methods

### Fungal and bacterial strains and growth conditions

The fungal strain *A. fumigatus* ATCC 46645 (American Type Culture Collection, LGC Standards) was used for all experiments. Conidia and germ tubes were prepared as previously described^24^. Germ tubes were inactivated by incubation in 100% ethanol for 45 min. Wild-type *C. albicans* SC5314 was maintained as previously described^29^. For experiments, colonies were transferred to M199 medium (PAA), pH 4 and cultured at 30 °C to stationary phase. Germ tubes were induced by culturing in M199 medium, pH 8 for one hour at 37 °C. *E.coli* ATCC25922 was cultured in LB (1% tryptone, 0.5% yeast extract, 1% sodium chloride, Carl Roth) medium. *C. albicans* and *E. coli* cells were inactivated by washing them in phosphate-buffered saline (PBS) followed by incubation in PBS containing 0.1% thimerosal (Sigma-Aldrich) for one hour at 37 °C with shaking. Afterwards, cells were washed 5 times and then re-suspended in RPMI-1640. Killing of *C. albicans* and *E. coli* cells was confirmed by plate counts on YPD (2% D-glucose, 1% peptone, 5% yeast extract, Roth) or LB agar plates, respectively.

### Generation of Monocyte-derived DCs

DCs were generated from PBMCs as previously described^24^. Briefly, PBMCs were isolated from healthy volunteers by ficoll (Bicoll Seperation, Biochrom AG) density gradient centrifugation. Magnetic activated cell sorting with paramagnetic CD14-beads (Miltenyi Biotec) was used to further separate monocytes. Monocyte-derived dendritic cells (DCs) were generated in RPMI-1640 supplemented with 10% FBS (Sigma-Aldrich), 120 mg/l Refobacin (Merck), 10 ng/ml IL-4 (Miltenyi Biotec) and 100 ng/ml GM-CSF (Bayer Healthcare) for 5 to 6 days.

Co-cultivation experiments of DCs with *A. fumigatus* or *C. albicans* and *E.coli* were performed on day 6 with a multiplicity of infection (MOI) of 1 in a concentration of 1*10^6^/ml. LPS (1 μg/ml, Sigma-Aldrich), LPS ultra-pure (1 μg/ml), zymosan depleted (100 μg/ml), Pam3CSK4 (100 ng/ml) (Invivogen) were used in the indicated concentrations.

### RNA interference

All RNA interference experiments were performed as previously described^24^. Briefly, DCs were electroporated (EPI 2500, Dr. L. Fischer) with either short interfering double stranded *KLF4*-siRNA, *CLEC7A*-siRNA or non-silencing, random RNA (Qiagen) at 340 V for 10 ms on day 5 after isolation and then incubated at 37 °C and 5% CO_2_ for 24 h in culture medium.

### RNA isolation

RNA was isolated by using RNeasy Mini Kit (Qiagen) according to manufacturer’s instructions. RNA quantity was determined using a Nanodrop 1000 (Thermo Scientific).

### Real Time RT-PCR

cDNA was generated from 100 ng template mRNA by First Strand cDNA synthesis Kit (Thermo Fisher Scientific). Quantitative real-time PCR was performed on a StepOne Plus (Applied Biosystem) with iTaq Universal SBYR Green Supermix (BioRad) and the primers for ***KLF4***(NM_004235.4) fw:TACCAAGAGCTCATGCCACC, rv: CGCGTAATCACAAGTGTGGG, ***IL6***(NM_000600.3) fw: AAAGAGGCACTGGCAGAAAA, rv: TTTCACCAGGCAAGTCTCCT, ***CCL2*** (NM_002982.3) fw:CCCCAGTCACCTGCTGTTAT, rv: AGATCTCCTTGGCCACAATG,***TNF***(NM_000594.2) fw TGCTTGTTCCTCAGCCTCTT, rv: TGGGCTACAGGCTTGTCACT***,CXCL10***(NM_001565.3)fw:CCACGTGTTGAGATCATTGC,rv:ATTTTGCTCCCCTCTGGTTT,***IL10***(NM_000572.2)fw:TTACCTGGAGGAGGTGATGC,rv:GGCCTTGCTCTTGTTTTCAC,**IL1B** (NM_000576.2)fw:GGACAAGCTGAGGAAGATG, rv:TCGTTATCCCATGTGTCGAA, **RANTES** (NM_002985.2)fw:GAGGCTTCCCCTCACTATCC, rv:CTCAAGTGATCCACCCACCT ***ALAS1***(NM_000688.5) fw: GGCAGCACAGATGAATCAGA, rv: CCTCCATCGGTTTTCACACT (Sigma-Aldrich) were used in a concentration of 0.5 μM at a total reaction volume of 20 μl.

### Transcriptome Analysis

DCs of four independent donors were harvested after six hours co-culture with *A. fumigatus*, *C. albicans* and LPS and analysed with Affymetrix whole genome expression arrays. RNA samples had a RNA integrity number (RIN) above 8 determined by Agilent Bioanalyzer. RNA samples were hybridized to an Affymetrix HG-U219 array plate. Scanned images were subjected to visual inspection to control for hybridization artefacts and proper grid alignment; these were analysed with AGCC 3.0 (Affymetrix) to generate CEL files. The resulting files, containing a single intensity value for each probe region delineated by a grid on each array image, were imported into Partek Genomics Suite (version 6.6, Partek, St. Louis, MO) for probe set summarization and statistical analysis. Model based Robust Multichip Analysis was performed for probe set summarization to obtain a single intensity value representing transcript abundance for each probe set and thus enable comparisons between arrays, by normalizing and logarithmically transforming array data and stabilizing variance across the arrays. Microarray data has been deposited in the GEO NCBI database (accession code GSE69723).

The R package limma was used to compare the expression of the probe sets between the conditions^30^,^31^. A probe set was regarded as significantly differentially expressed; if it has a False-Discovery-Rate (FDR) adjusted p-value < 0.01 a fold-change |FC| ≥ 2 of the mean transcript abundance^32^. Arrays were validated by RT-PCR *of IL6, KLF4, SYK, CCL5* and *IL1B. ALAS1* served as reference gene (not shown). Hierarchical clustering was based on the Spearman correlation of the samples, considering only the differentially expressed genes. The hclust function of R was used with “average linkage” as agglomeration method.

For the Gene Ontology (GO) analysis, the tool GOrilla^33^ was used to find overrepresented GO terms. Terms with an FDR adjusted p-value < 0.001 were considered as significantly enriched.

### Western Blotting

Cells were re-suspended in lysis buffer (6.65 M Urea (Sigma-Aldrich), 10% Glycerin, 1% SDS, 10 mM Tris (Carl Roth GmbH), pH 6.8), sonicated for 15 s (UP50H, Hielscher) and proteins quantified using the DC Protein Assay (BioRad). Cell lysates were subjected to sodium dodecyl sulfate polyacrylamide gel electrophoresis (SDS-PAGE) on 12% (w/v) gels and transferred to nitrocellulose membranes. Membranes were incubated with rabbit anti-KLF4 (Cell Signaling) and mouse anti-β-Actin (Sigma-Aldrich) antibodies over night at 4 °C or for 2 h at RT, respectively. Horseradish-conjugated anti-rabbit or anti-mouse IgG antibodies (Cell Signaling) were used as secondary antibodies. Signals were visualized by ECL reaction with Clarity ECL Western Substrate (BioRad) for KLF4 and Pierce ECL Western Blotting substrate (Thermo Scientific) for β-Actin and analyzed using ImageJ software (ImageJ version 1.47, National Institutes of Health).

### Quantification of secreted proteins

The concentrations of TNF-α, IL-1α, IL-1β, IL-10, IP-10 (CXCL10), MIP-3α (CCL20), IFN-gamma, IL-12p70, IL-15, IL-18, IL-2 [by Milliplex MAP, Millipore] and MCP-1 (CCL2), MIP-1α (CCL3), RANTES (CCL5) [by ProcartaPlex, eBioscience] in cell-free supernatants were determined by performing multiplex ELISA assays according to the instructions from the manufacturers.

IL-6 was analyzed using an in-house ELISA assay. In brief, 96-well medium-binding microplates were coated with 2 μg/ml IL-6 capture antibody (R&D Systems) in PBS overnight. All washing steps were performed with PBS + 0.05% Tween-20. Blocking of non-specific binding was achieved by incubation with PBS containing 1% BSA, 5% Succrose and 0.05% Sodium trinitride. Recombinant human IL-6 (R&D Systems) in PBS with 0.1% BSA served as standard. The biotinylated IL-6 detection antibody (0.1 μg/ml, R&D Systems) was diluted in 20 mM Trizma base, 150 mM sodium chloride and 0.1% BSA. HRP Streptavidin (Biolegend) was used in 1:1000 dilutions in PBS + 1% BSA. TMB solution was ordered at Biolegend. 1M hydrochloric acid served as stopping solution. Absorbance was measured at 450 nm at a spectrophotometer (Tecan).

### Statistics

All experiments were performed in sets of at least three independent experiments and the generated data was statistically analyzed by paired, two-tailed Student’s t-test (GraphPad Prism 5, GraphPad Software Inc.). Statistical values of p < 0.05 (*), 0.01 (**) and 0.001 (***) were considered significant.

### Ethical Approval

All methods were carried out in accordance with the approved guidelines #302/12 of the Ethical Committee of the University of Wuerzburg. Informed consent was written and provided by all study participants. Data analysis was conducted anonymously.

## Additional Information

**How to cite this article**: Czakai, K. *et al*. Krüppel-like Factor 4 modulates interleukin-6 release in human dendritic cells after *in vitro* stimulation with *Aspergillus fumigatus and Candida albicans.* *Sci. Rep.* **6**, 27990; doi: 10.1038/srep27990 (2016).

## Supplementary Material

Supplementary Information
