# Supplementary material for: Krüppel-like Factor 4 modulates interleukin-6 release in human dendritic cells after in vitro stimulation with Aspergillus fumigatus and Candida albicans
Source: Sci Rep. 2016 Jun 27;6:27990. doi: 10.1038/srep27990 (PMC4921831; doi:10.1038/srep27990)

**Krüppel-like Factor 4 modulates interleukin-6 release in human dendritic cells after *in vitro* stimulation with *Aspergillus fumigatus* and *Candida albicans***

# **Kristin Czakai^1^, Ines Leonhardt^2^, Andreas Dix^3^, Michael Bonin^4^, Joerg Linde^3^, Hermann Einsele^1^, Oliver Kurzai^2^, Jürgen Loeffler^1^***

^1^University Hospital of Würzburg, Department of Internal Medicine II, WÜ4i, Würzburg, Germany

^2^Septomics Research Centre, Friedrich Schiller University and Leibniz Institute for Natural Product Research and Infection Biology - Hans-Knöll-Institute (HKI), Jena, Germany.

**^3^**Systems Biology/Bioinformatics, Leibniz Institute for Natural Product Research and Infection Biology – Hans-Knöll-Institute, Jena, Germany

**^4^**Institute of Medical Genetics and Applied Genomics, University of Tübingen, Tübingen, Germany

**Supplementary Data**

Figure S1 Hierarchical clustering of differentially expressed genes. DCs were either stimulated with *A. fumigatus* germ tubes (Af), *C.* *albicans* germ tubes (Ca) (both MOI 1) or LPS (1 µg/ml) in four independent experiments for 6 h. Symbols indicate the different experiments. Affymetrix whole genome expression arrays (HGU-219) were performed. mRNAs with fold changes > 2 and a p-value < 0.01 were considered to be differentially regulated.

Figure S2 KLF4 was significantly down-regulated by electroporation with siKLF4 RNA. DCs were transfected by electroporation with either non-silencing siRNA control (ns) or siRNA targeting KLF4 (siKLF4) in four independent experiments. A) 24 h after electroporation, KLF4 mRNA was quantified by quantitative real-time PCR. ALAS1 served as housekeeping gene. B) Total cellular extracts of electroporated DCs, co-cultivated with *A. fumigatus* (Af) and *C. albicans* (Ca) (MOI 1) of LPS for 6 h were analyzed by western blotting. Cells were either electroporated with specific KLF4 siRNA (black bars) or non-silencing control (white bars). Protein levels were quantified relative to β-Actin. Significant changes in protein levels are indicated with asterisks (** p<0.01, *** p<0.001 Student’s paired t-test) Data is illustrated as mean plus standard error of the mean (SEM).

Figure S3 Target gene analysis of DCs stimulated with LPS in the absence of KLF4. DCs were transfected by electroporation with either non-silencing siRNA (white bars) or with siRNA targeting KLF4 (black bars). 24h after electroporation, DCs were stimulated with LPS (1 µg/ml) for 6 h. mRNA level were quantified by real-time PCR relative to non-silencing control. (* p<0.05, Student’s paired t-test) Data is illustrated as mean plus SEM.

Figure S4 KLF4 dependent cytokine regulation. DCs were transfected by electroporation with either non-silencing siRNA (white bars) or with siRNA targeting KLF4 (black bars). 24h after electroporation, DCs were stimulated with LPS (1µg/ml) *A. fumigatus* (Af) *C. albicans* (Ca) (both MOI 1) or left untreated for 6 h. Zytokine secretion was quantified by enzyme linked immunosorbent assay. Data is illustrated as mean plus SEM of 4 independent experiments (Student’s paired t-test).

**Figure S1**

**Figure S2**


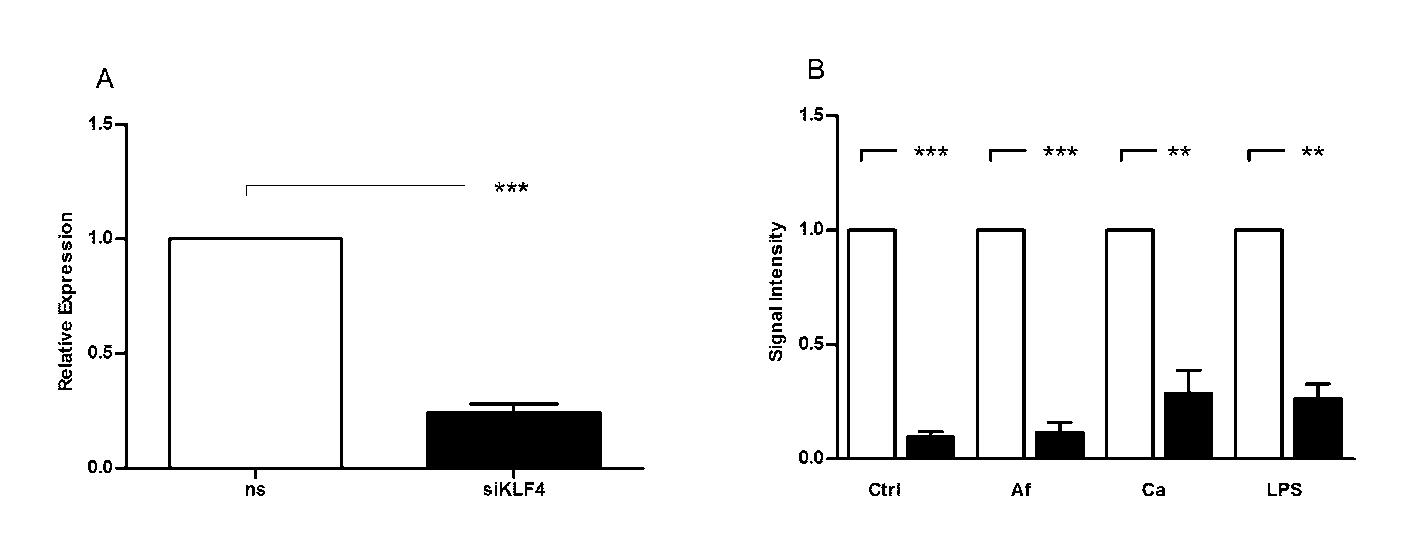


**Figure S3**


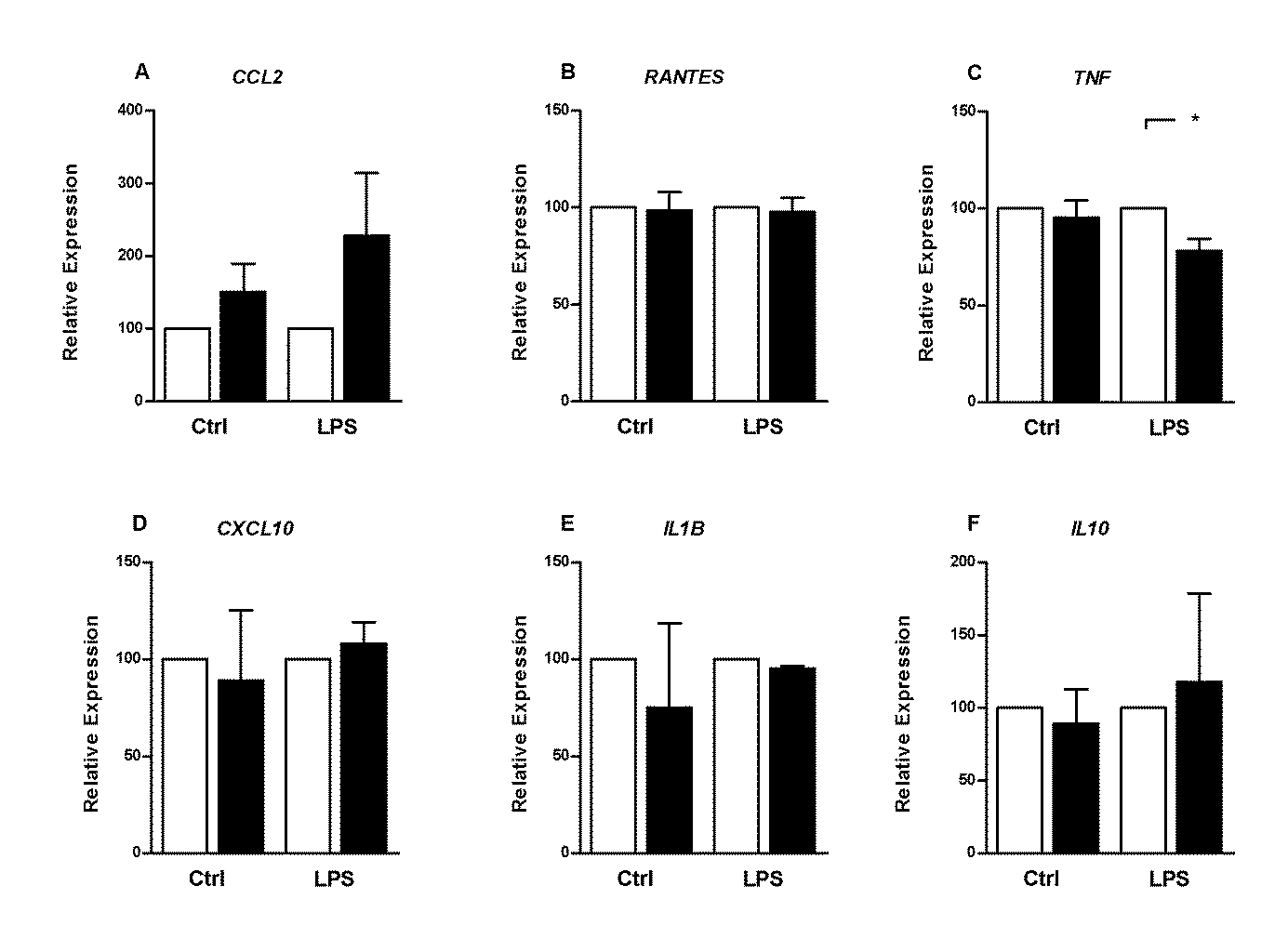


**Figure S4**


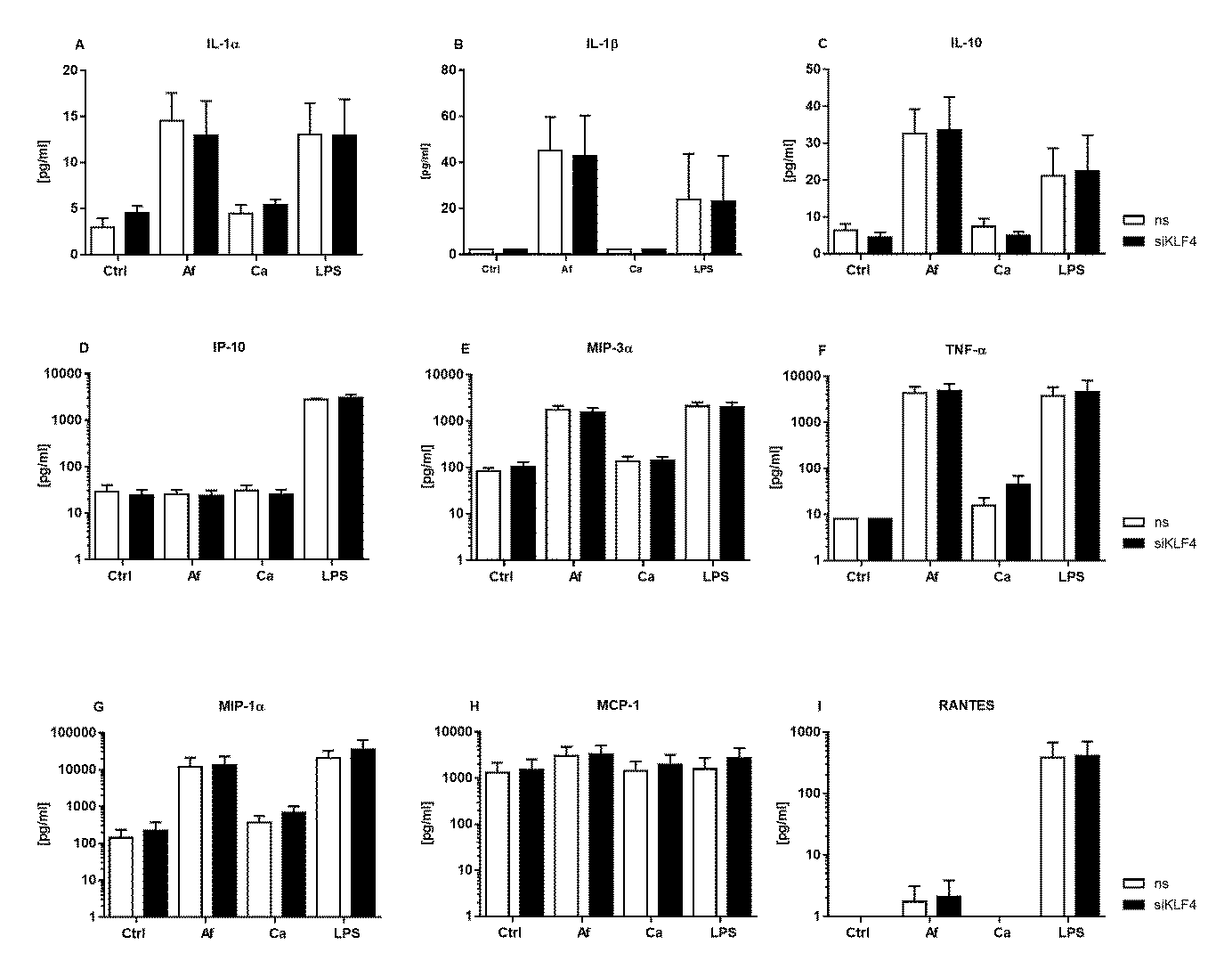

Supplement: Supplementary Information [file srep27990-s1.doc]
